# Vitamin D Signaling in Myogenesis: Potential for Treatment of Sarcopenia

**DOI:** 10.1155/2014/121254

**Published:** 2014-06-30

**Authors:** Akira Wagatsuma, Kunihiro Sakuma

**Affiliations:** ^1^Graduate School of Information Science and Technology, The University of Tokyo, 7-3-1 Hongo, Bunkyo-ku, Tokyo 113-8656, Japan; ^2^Research Center for Physical Fitness, Sports and Health, Toyohashi University of Technology, 1-1 Hibarigaoka, Tempaku-cho, Toyohashi 441-8580, Japan

## Abstract

Muscle mass and strength progressively decrease with age, which results in a condition known as sarcopenia. Sarcopenia would lead to physical disability, poor quality of life, and death. Therefore, much is expected of an effective intervention for sarcopenia. Epidemiologic, clinical, and laboratory evidence suggest an effect of vitamin D on muscle function. However, the precise molecular and cellular mechanisms remain to be elucidated. Recent studies suggest that vitamin D receptor (VDR) might be expressed in muscle fibers and vitamin D signaling via VDR plays a role in the regulation of myoblast proliferation and differentiation. Understanding how vitamin D signaling contributes to myogenesis will provide a valuable insight into an effective nutritional strategy to moderate sarcopenia. Here we will summarize the current knowledge about the effect of vitamin D on skeletal muscle and myogenic cells and discuss the potential for treatment of sarcopenia.

## 1. Introduction

Muscle wasting is observed in various disease states, in conditions of reduced neuromuscular activity, with ageing. Age-related muscle wasting is referred to as “sarcopenia” coined by Irwin H. Rosenberg from the Greek words sarx (meaning flesh) and penia (meaning loss) [1, 2]. There has been no consensus about definition of sarcopenia suitable for use in research and clinical practice [3]. Therefore, some studies [4, 5] suggest a working definition of sarcopenia: sarcopenia is a syndrome characterized by progressive and generalized loss of skeletal muscle mass and strength with a risk of adverse outcomes such as physical disability, poor quality of life, and death. Sarcopenia is characterized by the fact that it progresses very slowly throughout several decades. Muscle mass fairly consistently decreases at a rate of approximately 0.5–1%/year beginning at 40 years of age [6, 7] and the rate dramatically accelerates after the age of 65 years [8]. Muscle strength appears to decline more rapidly than muscle mass. Muscle strength declines at a rate of 3-4% per year in men and 2.5–3% per year in women aged 75 years [9]. Although the precise molecular and cellular mechanisms underlying age-related loss of muscle mass and strength have remained unknown [10, 11], multiple contributing factors have been proposed. The development and progress of sarcopenia have been thought to be mediated by the combination of these contributing factors.

Based on large-scale studies [12–16], on average, it is estimated that the prevalence of sarcopenia reaches 5–13% in those aged 60–70 years and ranges from 11 to 50% in those aged over 80 years [17]. In USA in 2000, it was estimated that direct healthcare costs related to sarcopenia were $18.5 billion ($10.8 billion in men, $7.7 billion in women), which represented approximately 1.5% of total healthcare expenditures for that year [18]. Globally, the number of people aged over 60 years is 600 million in the year 2000 [19]. It is predicted that people aged over 65 years will double by 2020 and will triple by 2050 [20]. Therefore, sarcopenia is being recognized as not only a serious healthcare problem but also a social problem. Much is expected of an effective intervention for sarcopenia. Nutritional interventions would be a promising candidate in combating sarcopenia.

Epidemiologic, clinical, and laboratory evidence provide an effect of vitamin D on muscle function. Numerous studies have investigated the effect of vitamin D supplementation on muscle strength and physical performance in elderly people. However, the precise molecular and cellular mechanisms remain to be elucidated. Immunohistochemical studies have demonstrated that vitamin D receptor (VDR) might be localized in human muscle fibers [21–23] with some contradictions [24, 25]. In addition, recent studies have reported that vitamin D signaling via VDR plays a role in the regulation of myoblast proliferation and differentiation [26–32]. Understanding how vitamin D signaling contributes to myogenesis will provide a valuable insight into an effective nutritional strategy to moderate sarcopenia. Here we will summarize the current knowledge about effect of vitamin D on skeletal muscle and myogenic cells and discuss the potential for treatment of sarcopenia.

## 2. Vitamin D Signaling Pathways: Genomic and Nongenomic Pathways

Vitamin D signaling has been extensively investigated in a variety of cell types. During the past two decades, considerable progress has been made in understanding the action of 1*α*,25-dihydroxyvitamin D_3_ [1*α*,25(OH)_2_D_3_] on myogenic cells. The biological effect of 1*α*,25(OH)_2_D_3_ is exerted through genomic or nongenomic mechanisms (for reviews see [33–35]). Better understanding of the molecular and cellular mechanisms of vitamin D action on skeletal muscle will enable us to develop an effective intervention for sarcopenia. We will focus on 1*α*,25(OH)_2_D_3_ signaling via VDR in genomic and nongenomic mechanism related to myogenic cells, although rapid alteration in intracellular calcium, which is nongenomically regulated by 1*α*,25(OH)_2_D_3_, has been well demonstrated both* in vivo* and* in vitro*; for details, excellent review article is already available on this subject [36].

An active form, 1*α*,25(OH)_2_D_3_, acts by binding to VDR [33]. The binding affinity of 25(OH)D_3_ for human vitamin D receptor (VDR) is approximately 500 times less than that of 1*α*,25(OH)_2_D_3_ but the circulating level of 25(OH)D_3_ is approximately 1000 times higher than that of 1*α*,25(OH)_2_D_3_ [37, 38]. In genomic mechanism, 1*α*,25(OH)_2_D_3_ binds to VDR and is transported to the nucleus [35]. VDR is heterodimerized with 9-*cis*-retinoic acid receptor (RXR) and VDR:RXR complex modulates gene expression via binding to specific target gene promoter regions, known as vitamin D response elements (VDREs), to activate or suppress their expression [35]. In general, VDREs possess either a direct repeat of two hexanucleotide half-elements with a spacer of three nucleotides (DR3) or an everted repeat of two half-elements with a spacer of six nucleotides (ER6) motif, with DR3s being the most common [39]. Wang et al. [40] investigated direct 1*α*,25(OH)_2_D_3_-target genes on a large scale by using a combined approach of microarray analysis and in silico genome-wide screens for DR3 and ER6-type VDREs. Microarray analyses, performed with RNA from human SCC25 cells treated with 1*α*,25(OH)_2_D_3_ and cycloheximide, an inhibitor of protein synthesis, revealed 913 regulated genes [40]. Of the 913 genes, 734 genes were induced and 179 genes were repressed by treatment of 1*α*,25(OH)_2_D_3_ [40]. In addition, a screening of the mouse genome identified more than 3000 conserved VDREs, and 158 human genes containing conserved elements were 1*α*,25(OH)_2_D_3_-regulated on microarrays [40]. These results support their broad physiological actions of 1*α*,25(OH)_2_D_3_ in a variety of cell types.

With respect to several genes related to myogenesis, we will describe them in more detail. For example, 1*α*,25(OH)_2_D_3_ induced expression of the gene encoding* Foxo1* [40], which is a member of the FOXO subfamily of forkhead/winged helix family of transcription factors, governs muscle growth, metabolism, and myoblast differentiation. When transfected C2C12 cells with adenoviral vector encoded a constitutively active Foxo1 mutant, they effectively blocked myoblast differentiation [47]. This was partly rescued by inhibition of Notch signaling [47], which inhibits myoblast differentiation [48]. In addition, loss of Foxo1 function precluded Notch signaling-mediated inhibition of myoblast differentiation [47]. To elucidate the possible role of Notch signaling in Foxo1-mediated inhibition of myoblast differentiation, by combining coculture system, transfection assay, chromatin immunoprecipitation assay, and short interfering RNA (siRNA) technology, authors showed that Foxo1 physically and functionally interacted with Notch by promoting corepressor clearance from DNA binding protein, CSL [CBF1/RBPjk/Su(H)/Lag-1], leading to inhibition of myoblast differentiation through activation of Notch target genes [47]. Another gene, Id (inhibitor of differentiation) gene, is also known target of 1*α*,25(OH)_2_D_3_ [49]. Id mRNA was constitutively expressed in rat osteoblastic osteosarcoma ROS17/2.8 cells and its level was transcriptionally suppressed by 1*α*,25(OH)_2_D_3_ [50]. 1*α*,25(OH)_2_D_3_ exerted its negative effect on Id1 gene transcription via the 57 bp upstream response sequence (−1146/−1090) [49]. Id proteins (Id1, Id2, Id3, and Id4) dimerize and neutralize the transcriptional activity of basic helix-loop-helix (bHLH) proteins [51]. It has been shown that Id inhibits MyoD activity either by forming transcriptionally inactive complexes of MyoD-Id or by forming heterodimers with E-proteins and effectively blocking the formation of active MyoD/E-protein complexes [52]. At this time, there are only limited data available on Id expression and vitamin D during muscle development. For example, in VDR knockout mice with abnormal muscle development, there were no differences in expression levels of Id1 and Id2 [46]. Therefore, we cannot conclude whether vitamin D regulates myogenesis by modulating Id expression.

A nongenomic response to 1*α*,25(OH)_2_D_3_ is characterized by a rapid (the seconds to minutes range) activation of signaling cascades and an insensitivity to inhibitors of transcription and protein synthesis [34]. The rapid response to 1*α*,25(OH)_2_D_3_ has been hypothesized to elicit the classic VDR translocation to the plasma membrane. When treating chick myoblasts with 1*α*,25(OH)_2_D_3_, translocation of VDR from the nucleus to the plasma membrane rapidly occurred within 5 min after the addition of 1*α*,25(OH)_2_D_3_ [53]. This translocation was blocked by colchicine, suggesting the possible role of the intracellular microtubular transport system in the distribution of VDR [53]. The VDR translocation appears to depend on intact caveolae that are specialized plasmalemmal microdomains originally studied in numerous cell types for their involvement in the transcytosis of macromolecules [54]. Confocal microscopy revealed that 1*α*,25(OH)_2_D_3_-induced VDR translocation to the plasma membrane was abolished by methyl-beta-cyclodextrin, a reagent to disrupt the caveolae structure [55]. Both disruption of caveolae and siRNA-mediated silencing of caveolin-1 suppressed 1*α*,25(OH)_2_D_3_-dependent activation of protooncogene c-Src (cellular Src) with tyrosine-specific protein kinase activity [55]. Immunocytochemical analysis provided evidence that caveolin-1 colocalized with c-Src near the plasma membrane under basal conditions [55]. When treated with 1*α*,25(OH)_2_D_3_, the colocalization of caveolin-1 and c-Src was disrupted and they were redistributed into cytoplasm and nucleus [55]. On the basis of these results, it can be hypothesized that (1) interaction caveolin-1/c-Src inactivates the kinase under basal conditions and (2) when 1*α*,25(OH)_2_D_3_ stimulates VDR translocation to the plasma membrane, it dissociates the caveolin-1/c-Src complex allowing c-Src activation [55]. Non-genomic action of 1*α*,25(OH)_2_D_3_ might be required for a reciprocal interaction between c-Src and caveoline-1. Besides the classical VDR, it has been identified as a potential candidate as an alternate membrane-associated receptor for 1*α*,25(OH)_2_D_3_: 1,25D_3_-MARRS (membrane-associated, rapid response steroid binding) also known as ERp57, GRp58, ERp60, and Pdia3 [56]. Since 1,25D_3_-MARRS has been shown to function in various cell types [57], it also may potentially mediate vitamin D signaling in myogenic cells.

The c-Src tyrosine kinase induced by 1*α*,25(OH)_2_D_3_ is required for activation of mitogen-activated protein kinases (MAPKs), ERK1/2 (extracellular signal-regulated kinase 1/2) [58], and p38 [59]. 1*α*,25(OH)_2_D_3_ rapidly promoted phosphorylation of ERK1/2 through c-Src activation [58], Raf-1/Ras/MEK (MAPK/ERK kinase), and PKC*α* (protein kinase C alpha) [60]. In addition to ERK1/2 activation, 1*α*,25(OH)_2_D_3_ rapidly stimulated MKK3/MKK6 (mitogen-activated protein kinase kinases 3/6)/p38 MAPK through c-Src activation [59]. Although another MAPK family member, JNK1/2 (c-Jun NH2-terminal kinase 1/2), was also activated by 1*α*,25(OH)_2_D_3_ [59], an upstream mediator of 1*α*,25(OH)_2_D_3_-dependent JNK1/2 activation was characterized less than that of ERK/1/2 and p38. The molecular links between JNK and c-Src have been shown in* Drosophila melanogaster*. The JNK homolog Basket (Bsk) is required for epidermal closure [61]. Src42A, a* Drosophila* c-Src protooncogene homolog functions in epidermal closure during both embryogenesis and metamorphosis [61]. The severity of the epidermal closure defect in the Src42A mutant depended on the Bsk activity. These results suggest the possibility that JNK activation in mammals may also be required for Src tyrosine kinase activity. These MAPK signaling pathways have been shown to contribute to myogenesis [62–65]. For example, inactivation of the Raf-1/MEK1/2/ERK1/2 pathway in MM14 cells through the overexpression of dominant negative mutants of Raf-1 blocked ERK1/2 activity and prevented myoblast proliferation [62]. Pharmacological blockade of p38*α*/*β* kinases by SB203580 inhibited myoblast differentiation [63–65]. JNK was involved in regulating myostatin signaling [66], which is known as a member of tumor growth factor *β* family and functions as a negative regulator of muscle growth [67]. MAPK signaling pathways function at different stages of myogenesis.

Apart from MAPKs, PI3K (phosphatidyl inositol 3-kinase)/Akt signaling pathway, which is essential for initiation of myoblast differentiation [68], also seems to be activated by 1*α*,25(OH)_2_D_3_. After exposure to 1*α*,25(OH)_2_D_3_, Akt phosphorylation was enhanced through PI3K in C2C12 cells [68]. Intriguingly, suppression of c-Src activity by PP2, a specific inhibitor for all members of the Src family, and knockdown of c-Src expression by siRNA decreased Akt phosphorylation in 1*α*,25(OH)_2_D_3_-treated C2C12 cells [28]. In addition, when treating C2C12 cells with 1*α*,25(OH)_2_D_3_ in the presence of U0126 or SB203580 to inhibit ERK1/2 and p38 MAPK, respectively, SB203580 but not U0126 markedly blocked both basal and 1*α*,25(OH)_2_D_3_-induced Akt phosphorylation. These results suggest that 1*α*,25(OH)_2_D_3_-induced Akt phosphorylation may occur through c-Src and p38 MAPK [28]. Taken together, 1*α*,25(OH)_2_D_3_ can simultaneously activate multiple signaling pathways in myogenic cells but their relative contribution to myogenesis remains to be established.

## 3. Effects of Ageing on Serum Concentration of Vitamin D, Muscle Morphology, and Muscle Fiber Type

Vitamin D status varies with age [69]. Serum levels of 25(OH)D_3_ are qualitatively categorized as deficiency (<20 ng/L or <50 nM), insufficiency (21–29 ng/L or 50–75 nM), and normal (30 ng/L or >75 nM) [70]. van der Wielen et al. [69] measured wintertime serum 25(OH)D_3_ concentrations in 824 elderly people from 11 European countries [69]. They reported that 36% of men and 47% of women had 25(OH)D_3_ concentrations below 30 nM [69]. Vitamin D deficiency in elderly is thought to occur mainly due to restricted sunlight exposure, reduced dietary vitamin D intake, and decreased capacity of the skin to produce vitamin D [69]. MacLaughlin and Holick [71] examined the effects of ageing on the capacity of the skin to produce previtamin D3 in the skin by comparing young subjects (8 and 18 years old) with aged subjects (77 and 82 years old). They showed that ageing decreased the capacity less than half of young subjects [71], suggesting that elderly people are potentially at risk for vitamin D insufficiency/deficiency.

Vitamin D deficiency appears to be associated with changes in muscle morphology. For example, patients with osteomalacic myopathy associated with vitamin D deficiency show degenerative changes such as opaque fibers, ghost-like necrotic fibers, regenerating fibers, enlarged interfibrillar spaces, infiltration of fat, fibrosis, glycogen granules, and type II muscle fiber atrophy [72]. As is the case with vitamin D-deficient patients, it is well known that elderly people show aberrant muscle morphology. Scelsi et al. [73] performed histochemical and ultrastructure analysis using biopsies taken from the vastus lateralis of healthy sedentary men and women aged 65–89 years. They observed myofibrillar disorganization, streaming of Z-line, rod formation, intracellular lipid droplets, lysosomes, and type II muscle fiber atrophy [73]. The very elderly people had “flattened” or “crushed” shaped muscle fibers, whereas the young people had mature-appearing polygonal muscle fibers [74]. These aberrant changes were much more pronounced in the type II muscle fibers than in type I muscle fibers [74]. Although the precise mechanisms remain to be elucidated, it can be speculated that specific type II muscle fiber atrophy with ageing may be associated with a muscle fiber type-specific reduction in satellite cell content. Satellite cells are essential for normal muscle growth [75]. Verdijk et al. [76] examined whether satellite cells could specifically decrease in type II muscle fibers in the elderly people. Biopsies were taken from the vastus lateralis of elderly (average age: 76 years) and young (average age: 20 years) healthy males [76]. They found significant reduction in the proportion and mean cross-sectional area of the type II muscle fibers and the number of satellite cells per type II muscle fiber in elderly subjects compared to young subjects [76]. This study is the first to show type II muscle fiber atrophy in elderly people to be associated with a muscle fiber type-specific decline in satellite cell content. It remains unknown whether vitamin D supplementation specifically attenuates atrophy of type II muscle fibers with recruitment of satellite cell. Whether vitamin D has positive effects on myoblast proliferation and differentiation is currently under debate. Recent studies [27] suggest that vitamin D treatment enhances fast type (type IIa) MyHC expression in fully differentiated C2C12 myotubes. Type II muscle fibers contain type IIa MyHC [77]. Therefore, vitamin D could potentially contribute to the changes in phenotype of existing muscle fibers and/or the maintenance of type II muscle fibers.

## 4. Effects of Ageing on Expression of VDR

Bischoff-Ferrari et al. [22] investigated the effect of ageing on VDR expression in human skeletal muscle. Biopsies were taken from the gluteus medius of 20 female patients undergoing total hip arthroplasty (average age: 71.6 years) and from the transversospinalis muscle of 12 female patients with spinal operations (average age: 55.2 years). Immunohistochemical analysis revealed that the number of VDR-positive myonuclei decreased with ageing [22]. Importantly, VDR expression was not affected by 25(OH)D_3_ or 1*α*,25(OH)_2_D_3_ levels [22]. Buitrago et al. [78] showed that silencing of VDR expression in C2C12 myoblasts suppressed p38 MAPK phosphorylation and decreased ERK1/2 activation induced by 1*α*,25(OH)_2_D_3_. Tanaka et al. [31] demonstrated that knockdown of VDR expression resulted in downregulation of MyHC mRNA in differentiating C2C12 myoblasts when treated with 1*α*,25(OH)_2_D_3_. Therefore, it is possible that decreased expression of VDR observed in elderly people might reduce the functional response of the muscle fibers to 1*α*,25(OH)_2_D_3_.

## 5. Effects of Vitamin D Supplementation on Muscle Injury

The regenerative potential of skeletal muscle decreases with age [79–81]. Satellite cells are absolutely required for muscle regeneration [82]. Satellite cells are defined anatomically by their position beneath the basal lamina and adhered to muscle fibers [83]. They, traditionally considered as a population of skeletal muscle-specific committed progenitors, play a crucial role in the postnatal maintenance, repair, and regeneration [75]. Under normal physiological conditions, they remain in a quiescent and undifferentiated state [75, 84]. However, when skeletal muscle is damaged by unaccustomed exercise or mechanical trauma, they are activated to proliferate, differentiate, and fuse with the already existing muscle fibers or fuse to form new muscle fibers [75, 84]. Few studies have examined the effects of vitamin D treatment on muscle injury. Stratos et al. [85] investigated whether systemically applied vitamin D could restore muscle function and morphology after trauma. Rats were injected subcutaneously with 7-dehydrocholesterol (332,000 IU/kg) immediately after crush injury and muscle samples were collected at days 1, 4, 14, and 42 after injury [85]. Vitamin D treatment increased cell proliferation and inhibited occurrence of apoptosis at day 4 compared to control rats [85]. In addition, a faster recovery of contraction forces was observed at day 42 in vitamin D-treatment group compared to control group [85]. Notably, the number of satellite cells was not influenced by vitamin D [85], suggesting the possibility that vitamin D supplementation has relatively little effect on satellite cell function* in vivo*. It is necessary to scrutinize thoroughly efficacy, duration, optimal dose, and side effects in relation to vitamin D treatment. Srikuea et al. [29] demonstrated that VDR was highly expressed in the nuclei of regenerating muscle fibers, indicating a potential role for vitamin D in muscle regeneration following injury. Relationship of vitamin D signaling and myogenesis will be discussed below in Section 10.

## 6. Vitamin D and Type 2 Diabetes Mellitus

Although the incidence of type 2 diabetes mellitus increases with age [86], the precise underlying mechanisms are still not fully understood. Skeletal muscle is the primary target for insulin action and glucose disposal. Therefore, elderly people with excessive loss of muscle mass are at risk for development of type 2 diabetes mellitus [87]. Meta-analysis reveals that vitamin D supplementation has beneficial effects among patients with glucose intolerance or insulin resistance at baseline [88]. However, an explanation for the beneficial role of vitamin D supplementation in the lowering of glycemia in diabetes mellitus remains to be determined. Skeletal muscle can increase glucose uptake through insulin-dependent and muscle contraction-dependent mechanisms [89]. Insulin and muscle contractions stimulate glucose transport in skeletal muscle via translocation of intracellular glucose transporter type 4 (GLUT4) to the cell surface. Manna and Jain [90] examined the mechanism by which vitamin D supplementation regulates glucose metabolism in 3T3L1 adipocytes. When 3T3L1 adipocytes were treated with high glucose in the presence of 1*α*,25(OH)_2_D_3_, it increased expression of GLUT4 and its translocation to cell surface, glucose uptake, and glucose utilization [90]. 1*α*,25(OH)_2_D_3_ also enhanced cystathionine-*γ*-lyase (CSE) activation and H_2_S formation [90], which is an important signaling molecule produced mainly by CSE in the cardiovascular system [91]. Furthermore, the effect of 1*α*,25(OH)_2_D_3_ on GLUT4 translocation and glucose utilization was prevented by chemical inhibition or silencing of CSE [90]. In muscle cells, it is currently not known whether CSE may be associated with 1*α*,25(OH)_2_D_3_-induced glucose metabolism. Therefore, further studies are required to elucidate the physiological role of CSE in regulation of glucose metabolism in skeletal muscle. Tamilselvan et al. [92] examined the effect of calcitriol (1,25, dihydroxycholecalciferol) on the expression of VDR, insulin receptor (IR), and GLUT4 in L6 cells when exposed to high glucose and high insulin which mimics type 2 diabetic model [92]. Calcitriol partially restored VDR, IR, and GLUT4 expression in type 2 diabetic model [92], raising the possibility that vitamin D could contribute to improving insulin signaling in type 2 diabetes mellitus. In a recent study, the effect of 1*α*,25(OH)_2_D_3_ on glucose uptake in rat skeletal muscle is investigated [93]. 1*α*,25(OH)_2_D_3_ stimulated glucose uptake with increased expression of GLUT4 protein and enhanced translocation of GLUT4 to the plasma membrane not through PI3K-signaling pathway [93], which is essential for insulin-stimulated GLUT4 translocation and glucose transport [94]. In addition, 1*α*,25(OH)_2_D_3_-stimulated glucose uptake was suppressed concomitantly with downregulation of GLUT4 protein by treatment with cycloheximide [93], suggesting that it may be mediated by genomic signaling of vitamin D. Taken together, vitamin D may improve glucose metabolism in skeletal muscle by modulating GLUT4 expression and translocation through insulin-dependent and/or insulin-independent mechanisms.

## 7. Vitamin D Receptor Expression in Skeletal Muscle and Myogenic Cells

VDR is known to be expressed in a wide variety of tissues including bone, bronchus, intestine, kidney, mammary gland, pancreas, parathyroid, pituitary gland, prostate gland, spleen, testis, and thymus [25]. However, there has been still some controversy as to whether VDR is expressed in skeletal muscle [24, 29, 32]. For example, some studies have failed to detect VDR in skeletal muscle [24, 25, 95–97]; other studies have shown that VDR protein and/or mRNA are detectable in skeletal muscle [21–23, 29, 46, 98] and myogenic cells [26, 27, 29, 31, 32, 42, 45, 46, 53, 55, 98–104]. In brief, Wang et al. [105] call into question the specificity of various commercially available VDR antibodies. They systematically characterized these antibodies in terms of their specificity and immunosensitivity using negative control samples from VDR knockout mice [105]. They demonstrated that the mouse monoclonal VDR antibody against the C-terminus of human VDR, D-6 (Santa Cruz Biotechnology), possesses high specificity, high sensitivity, and versatility [105]. They showed that VDR protein was not detected in skeletal muscle by immunohistochemical analysis using this antibody and that VDR mRNA was detectable only at extremely low levels by quantitative RT-PCR assay [24]. By contrast, Kislinger et al. [106] used large scale gel-free tandem mass spectrometry to monitor global proteome alterations throughout the myogenic differentiation program in C2C12 cells. They observed upregulation of VDR protein during early stage of myoblast differentiation [106]. Srikuea et al. [29] provided strong evidence for the presence of VDR in myogenic cells, by combining immunoblot assay, immunocytochemical analysis, PCR-based cloning, and DNA sequencing to validate the expression of VDR in C2C12 cells. They showed that the full-length VDR mRNA transcript could be isolated from myoblasts and myotubes and VDR protein was primarily localized in the nucleus of myoblasts and in the cytoplasm of myotubes [29]. In addition, they examined the localization of VDR protein using a model of myogenesis* in vivo*. BaCl_2_ treatment was used to induce regeneration and immunohistochemical analysis was performed on sections from control and regenerating muscle. In control muscle, VDR was detected in muscle fibers but levels were very low, whereas in regenerating muscle, VDR expression was detected in the central nuclei of newly regenerating muscle fibers [29]. More recently, Girgis et al. [32] demonstrated that VDR protein was detectable in C2C12 myoblasts by immunoblot assay using VDR antibody (D-6). The discrepancy among studies may be explained, at least in part, by the difference in the expression of VDR during the stages of muscle development. For example, Endo et al. [46] reported that VDR mRNA was detected in skeletal muscle from 3-week-old wild-type mice but not 8-week-old wild-type mice. Wang and DeLuca [24] showed that VDR protein was undetectable in skeletal muscle from 6- to 7-week-old C57BL/6 mice. A similar result was also reported by Srikuea et al. [29] using 12-week-old C57BL/6 mice. Therefore, VDR expression may be dependent on the context of muscle development. It requires further clarification whether VDR is expressed in muscle fibers.

## 8. The Conversion of 25 (OH) **D**_ 3_ to 1**α**,25 **(OH)**_ 2_ **D**_**3**_ Might Occur in Myogenic Cells

Vitamin D, in the form of vitamin D_3_, is synthesized from 7-dehydrocholesterol in the skin through the action of ultraviolet irradiation [33]. Alternatively, vitamin D, in the form of either vitamin D_2_ or vitamin D_3_, can also be taken in the diet [33]. An active form, 1*α*,25(OH)_2_D_3_, is synthesized from vitamin D_3_ through two hydroxylation steps [33]. Vitamin D_3_ is converted to 25-hydroxyvitamin D_3_ [25(OH)D_3_] in the liver by 25-hydroxylases (encoded by the gene* CYP27A1*) [33]. The generated 25(OH)D_3_ is further hydroxylated to 1*α*,25(OH)_2_D_3_ by 25-hydroxyvitamin D_3_ 1*α*-hydroxylase (encoded by the gene* CYP27B1*) in the kidney [33]. However, CYP27B1 has been detected in various extrarenal tissues [107, 108], raising the possibility that 1*α*,25(OH)_2_D_3_ might be locally synthesized and activate VDR in myogenic cells [29, 32]. Inactive form of vitamin D_3_, 25(OH)D_3_, could inhibit cell proliferation in a similar manner to 1*α*,25(OH)_2_D_3_ [29, 32], indicating that the conversion of 25(OH)D_3_ to 1*α*,25(OH)_2_D_3_ by CYP27B1 occurs in myogenic cells. Girgis et al. [32] confirmed this possibility using luciferase reporter assay system that luciferase activity results from 1*α*,25(OH)_2_D_3_ binding to GAL-4-VDR and subsequent activation of the UASTK luciferase gene via its GAL4 promoter. They transfected C2C12 cells with GAL4-VDR (switch) and UASTK luciferase reporter with treatment of 25(OH)D_3_ and showed that luciferase activity increased in a dose-dependent manner, suggesting the conversion of 25(OH)D_3_ to 1*α*,25(OH)_2_D_3_ by CYP27B1 and the subsequent activation of luciferase expression via 1,25(OH)_2_D-bound GAL4-VDR [32]. Srikuea et al. [29] confirmed that C2C12 cells express the full-length* CYP27B1 *mRNA transcript and CYP27B1 protein could be detected in the cytoplasm of myoblasts, exhibiting partially overlapping with the mitochondria to which CYP27B1 has been reported to be typically localized [109]. Furthermore, they showed that siRNA-mediated knockdown of CYP27B1 could alleviate inhibitory effects of 25(OH)D_3_ on cell proliferation [29]. These observations provide direct evidence that CYP27B1 is biologically active in myogenic cells and mediates to convert 25(OH)D_3_ to 1*α*,25(OH)_2_D_3_. However, it should be noted that the agonistic action of 25(OH)D_3_ has been demonstrated in cells derived from CYP27B1 knockout mice [110]. Although further studies are needed to elucidate the basic mechanisms, locally synthesized 1*α*,25(OH)_2_D_3_ in myogenic cells might act through autocrine/paracrine mechanisms via VDR.

## 9. The Role of VDR in Muscle Development

Since the process of myogenesis has been extensively studied both* in vivo* and* in vitro*, substantial progress has been made in understanding the molecular and cellular mechanisms. The myogenic regulatory factors, a group of basic helix-loop-helix transcription factors, consisting of MyoD, Myf5, myogenin, and MRF4, play critical roles in myogenesis [111]. MyoD and Myf5 have redundant functions in myoblast specification [112, 113], whereas myogenin [114, 115] and either MyoD or MRF4 [116] are required for differentiation. These myogenic factors can form heterodimers in combination with less specific factors such as members of E12/E47 [117], which are generated by alternative splicing of the E2A gene [118], leading to activation of muscle-specific gene transcription [117].

VDR knockout mice model has provided insight into the possible physiological roles of vitamin D signaling via its receptor in muscle development [46]. VDR null mice recapitulate a human disease of vitamin D resistance, vitamin D-dependent rickets type II [119]. VDR null mice grow normally until weaning and thereafter develop various metabolic abnormalities including hypocalcemia, hypophosphatemia, secondary hyperparathyroidism, and bone deformity [46, 119]. Muscle fiber diameter of VDR null mice was approximately 20% smaller and fiber size was more variable than that of the wild-type mice at 3 weeks of age (before weaning). By 8 weeks of age, these morphological changes were more prominent in the VDR null mice compared to the wild-type mice, suggesting either a progressive nature of the abnormalities caused by the absence of VDR or additive effects of systemic metabolic changes already present at this age [46]. Although there are neither degenerative nor necrotic changes in VDR null mice, the aberrant myofibers were observed diffusely without any preference to type I or type II fibers [46]. Based on these results, they suggest that the absence of VDR induces these abnormalities probably in late stages of fiber maturation and/or in metabolism of mature muscle fibers. Tanaka et al. [31] showed that siRNA-mediated knockdown of VDR inhibited myotube formation concomitantly with downregulation of MyoD and myogenin using C2C12 and G8 cells. These results demonstrate that a substantial level of signaling via VDR is required for normal muscle development and myogenesis* in vitro*.

Furthermore, Myf5, myogenin, and E2A but not MyoD and MRF4 were aberrantly and persistently upregulated at the protein and/or mRNA levels in VDR null mice at 3 weeks of age [46]. Consistent with the deregulated expression of MRFs that control muscle phenotype, VDR null mice showed aberrantly increased expression of embryonic and neonatal MyHC isoforms but not type II (adult fast twitch) MyHC isoform [46]. These findings observed in VDR null mice may reflect compensatory response to a reduction in muscle fiber size. For example, it can be hypothesized that, in VDR null mice, satellite cells may be anomalously activated, proliferate, and differentiate to form new myonuclei that fuse with existing fibers to restore normal fiber size. Finally, they examined whether 1*α*,25(OH)_2_D_3_ could directly downregulate MRFs and neonatal MyHC gene expression in C2C12 myoblasts. 1*α*,25(OH)_2_D_3_ (10 nM) decreased the steady-state expression levels of these genes [46]. Overall, these results support a role of VDR in the regulation of muscle development, but the precise mechanisms remain to be elucidated and the interpretation is further complicated since negative vitamin D response elements [120–122] in the promoter region of genes encoding Myf5 and myogenin have not been identified.

## 10. Effects of 1**α**,25 **(OH)**_ 2_ **D**_ 3_ on Myoblast Proliferation and Differentiation

As referred to above, decline of intrinsic regenerative potential of skeletal muscle is a hallmark of ageing [79–81] and may be due to age-related changes in satellite cell function. If vitamin D treatment does lead to improvements in muscle function in elderly people, more attention should be directed to the effect of vitamin D_3_ on myoblast proliferation and differentiation. Research on effect of 1*α*,25(OH)_2_D_3_ on myogenesis has been performed using an* in vitro* cell culture system. The effects of 1*α*,25(OH)_2_D_3_ on myoblast proliferation and differentiation are summarized in Table 1. Early studies [41, 43] have reported that 1*α*,25(OH)_2_D_3_ stimulates proliferation of myogenic cells. Giuliani and Boland [41] reported that 1*α*,25(OH)_2_D_3_ (0.13 nM) increased cell density of chick myoblasts. Drittanti et al. [43] showed that 1*α*,25(OH)_2_D_3_ (0.1 nM) had biphasic effects on DNA synthesis. 1*α*,25(OH)_2_D_3_ exhibited a mitogenic effect in proliferating chick myoblasts followed by an inhibitory effect during the subsequent stage of myoblast differentiation. Capiati et al. [44] showed that 1*α*,25(OH)_2_D_3_ (1 nM) increases the rate of [^3^H] thymidine incorporation into DNA in chick myoblasts. In addition, they investigated the role of PKC in mediating the effect of 1*α*,25(OH)_2_D_3_ using a PKC inhibitor. PKC activity increased after treatment of 1*α*,25(OH)_2_D_3_ [44]. The specific PKC inhibitor, calphostin, suppressed 1*α*,25(OH)_2_D_3_ stimulation of DNA synthesis in proliferating myoblasts [44]. Finally, they examined 1*α*,25(OH)_2_D_3_-dependent changes in the expression of PKC isoforms *α*, *β*, *δ*, *ε*, and *ζ* [44]. They identified PKC*α* as main isoform correlated with the early stimulation of myoblast proliferation by 1*α*,25(OH)_2_D_3_ [44]. By contrast, several studies suggest that, overall, 1*α*,25(OH)_2_D_3_ or 25(OH)D_3_ appears to have antiproliferative effect on myogenic cells [26, 27, 29, 32, 42]. 1*α*,25(OH)_2_D_3_ (1–100 nM) inhibited proliferation of C2C12 myoblasts in a dose-dependent manner [27, 32] without inducing necrotic and apoptotic cell death [32]. Okuno et al. [27] showed that 1*α*,25(OH)_2_D_3_ arrested the cells in the G0/G1 phase concomitantly with induction of cyclin-dependent kinase (CDK) inhibitors, p21^WAF1/CIP1^ that facilitates cell cycle withdrawal [123] and p27^Kip1^ that inhibits a wide range of CDKs essential for cell cycle progression [124]. Girgis et al. [32] also reported the increased expression of genes involved in G0/G1 arrest including Rb (retinoblastoma protein) and ATM (ataxia telangiectasia mutated) and decreased expression of genes involved in G1/S transition, including c-myc (cellular myc) and cyclin-D1. In addition, they found reduced c-myc protein and hypophosphorylated Rb protein [32]. The active form, hypophosphorylated Rb, blocks entry into S-phase by inhibiting the E2F transcriptional program [125, 126]. In summary, the effects of 1*α*,25(OH)_2_D_3_ on myoblast proliferation remain inconclusive. The discrepancy may be due to the differences in the experimental settings. For example, different cell type (primary cells or immortalized cell lines): 1*α*,25(OH)_2_D_3_ concentration, serum concentration, duration of cell culture, and duration of treatment are employed. Further studies are needed to clarify the role of 1*α*,25(OH)_2_D_3_ on myoblast proliferation.

Some studies [43, 44] reported that 1*α*,25(OH)_2_D_3_ (0.1 or 1 nM) had inhibitory effects on DNA synthesis in differentiating chick myoblasts, with an increased MyHC expression, increased myofibrillar and microsomal protein synthesis, and an elevation of creatine kinase activity. Garcia et al. [26] reported that prolonged treatment of C2C12 myoblasts with 1*α*,25(OH)_2_D_3_ (100 nM) enhanced myoblast differentiation by inhibiting cell proliferation and modulating the expression of promyogenic and antimyogenic growth factors using a culture system without reducing serum concentration to initiate cell differentiation. They showed that 1*α*,25(OH)_2_D_3_ downregulated insulin-like growth factor-I (IGF-I) and myostatin expression and upregulates IGF-II and follistatin expression [26]. Follistatin antagonizes myostatin-mediated inhibition of myogenesis [127]. Intriguingly, inhibition of myostatin is characterized by increased expression of IGF-1 and IGF-II [128–133], which are known to be potent stimulus of myogenesis [134, 135]. Therefore, it can be hypothesized that 1*α*,25(OH)_2_D_3_ may contribute to myogenesis by inducing IGF-II expression through modulation of myostatin-follistatin system. It should be noted, however, that in these culture conditions, only small thin myotubes with few nuclei were observed on day 10 [26]. This may not recapitulate normal C2C12 myoblast differentiation as previously reported [136].

In general, C2C12 myoblasts normally proliferate and are mononucleated when kept subconfluently in high-mitogen medium (e.g., 10–20% fetal bovine serum). To initiate cell cycle exit and myogenic differentiation, by switching from high-mitogen medium to low-mitogen medium (e.g., 2% horse serum), they fuse and differentiate into postmitotic, elongated, and multinucleated myotubes. Using this C2C12 myoblast differentiation system, Buitrago et al. [28] showed that 1*α*,25(OH)_2_D_3_ (1 nM) enhanced the expression of MyHC and myogenin at 72 h after treatment. By contrast, Okuno et al. [27] investigated the effects of 1*α*,25(OH)_2_D_3_ (1–100 nM) on differentiating and differentiated stage of C2C12 myoblasts. In differentiating phase, 1*α*,25(OH)_2_D_3_ treatment downregulated the expression of neonatal myosin heavy chain and myogenin and inhibited myotube formation in a dose-dependent manner (1–100 nM) [27]. They showed that the expression of fast MyHC isoform increased when fully differentiated myotubes were treated with 1 and 10 nM 1*α*,25(OH)_2_D_3_ [27]. Girgis et al. [32] investigated the prolonged treatment of 1*α*,25(OH)_2_D_3_ (100 nM) on C2C12 myoblast differentiation. When myoblast was treated with 1*α*,25(OH)_2_D_3_ throughout myogenesis including proliferative, differentiating, and differentiated stages, myotube formation was delayed by day 10 concomitantly with downregulation of Myf5 and myogenin [32]. However, intriguingly, myotubes treated with 1*α*,25(OH)_2_D_3_ exhibit larger cell size than nontreated myotubes [32]. These results suggest that 1*α*,25(OH)_2_D_3_ may biphasically act in the process of early and late myoblast differentiation. Furthermore, they showed that the hypertrophic effect of 1*α*,25(OH)_2_D_3_ on myotubes is accompanied with downregulation of myostatin [32]. Several studies have provided evidence that myostatin acts as a negative regulator of the Akt/mammalian target of rapamycin (mTOR) signaling pathway [137–140], which plays a key role in the regulation of protein synthesis [141]. For example, Trendelenburg et al. [139] show that myostatin reduces Akt/mTOR signaling complex 1 (TORC1)/p70 S6 kinase (p70S6K) signaling, inhibiting myoblast differentiation and reducing myotube size. In addition, 1*α*,25(OH)_2_D_3_ induced Akt phosphorylation in differentiating C2C12 cells [28]. Intriguingly, 1*α*,25(OH)_2_D_3_ sensitizes the Akt/mTOR signaling pathway to the stimulating effect of leucine and insulin, resulting in a further activation of protein synthesis in C2C12 myotubes [104]. Taken together, 1*α*,25(OH)_2_D_3_ may have an anabolic effect on myotubes by modulating Akt/mTOR signaling probably through genomic and nongenomic mechanisms.

## 11. Conclusions

The randomized-controlled studies and meta-analysis support a role of vitamin D in improving the age-related decline in muscle function. However, the effect remains inconclusive. Girgis et al. [32] emphasize that large studies employing standardized, reproducible assessments of muscle strength and double-blinded treatment regimens are required to identify the effect of vitamin D supplementation on muscle function and guide the recommended level of vitamin D intake. Although it remains intensely debated whether VDR is expressed in skeletal muscle, research on VDR null mice provides insight into the physiological roles of vitamin D in muscle development and suggests that a substantial level of signaling via VDR is required for normal muscle growth. VDR expression seems to be affected by ageing, suggesting that this might reduce the functional response of the muscle fibers to vitamin D. Vitamin D appears to function in primary myoblasts and established myoblast cell lines. Despite limited evidence available at the time, vitamin D might have an anabolic effect on myotubes by modulating multiple intracellular signaling pathways probably through genomic and nongenomic mechanisms. However, not all studies support this result. Further studies on the potential impact of vitamin D on muscle morphology and function are required to develop the effective intervention for sarcopenia.

## Figures and Tables

**Table 1:** Effects of 1*α*,25(OH)_2_D_3_ on proliferation and differentiation in myogenic cells.

Muscle cell type	Concentration [1*α*,25(OH)_2_D_3_]	Proliferation	Differentiation	Method of VDR detection	Reference
Myoblast (chick)	0.13 nM	↑	↑	NI	Giuliani and Boland 1984 [41]
G8	3–300 nM	↓	NI	Equilibrium binding assay, chromatography	Simpson et al., 1985 [42]
Myoblast (chick)	0.1 nM	↑	↑	NI	Drittanti et al., 1989 [43]
Myoblast (chick)	1 nM	↑	↑	NI	Capiati et al., 1999 [44]
C2C12	1 nM	ND	NI	Immunoblot	Stio et al., 2002 [45]
C2C12	10 nM	NI	ND	RT-PCR	Endo et al., 2003 [46]
C2C12	100 nM	↓	↑	RT-PCR, immunoblot, and immunocytochemistry	Garcia et al., 2011 [26]
C2C12	1–100 nM	↓	↓	RT-PCR	Okuno et al., 2012 [27]
C2C12	1 nM	↑	↑	NI	Buitrago et al., 2012 [28]
C2C12	20 nM 2 *μ*M [25(OH)D_3_]	↓	↓	RT-PCR, PCR cloning, DNA sequencing, immunocytochemistry, and immunoblot	Srikuea et al., 2012 [29]
C2C12	0.1 pM–10 *μ*M	NI	↓	NI	Ryan et al., 2013 [30]
C2C12, G8	1–100 nM	NI	↓	RT-PCR	Tanaka et al., 2013 [31]
C2C12	1–100 nM 1–100 nM [25(OH)D_3_]	↓	↓	RT-PCR, immunoblot	Girgis et al., 2014 [32]

Promote (↑); inhibit (↓); no difference between vehicle and treatment (ND); not investigated (NI).
